# Spermidine Suppressed the Inhibitory Effects of Polyamines Inhibitors Combination in Maize (*Zea mays* L.) Seedlings under Chilling Stress

**DOI:** 10.3390/plants10112421

**Published:** 2021-11-10

**Authors:** Canhong Gao, Mohamed S. Sheteiwy, Chen Lin, Yajing Guan, Zaid Ulhassan, Jin Hu

**Affiliations:** 1 College of Agriculture, Anhui Agricultural University, Hefei 230036, China; gaocanhong@163.com; 2 Seed Science Center, College of Agriculture and Biotechnology, Zhejiang University, Hangzhou 310058, China; lincheng837243270@163.com; 3 Department of Agronomy, Faculty of Agriculture, Mansoura University, Mansoura 35516, Egypt; salahco_2010@yahoo.com; 4 Laboratory of Spectroscopy Sensing, Institute of Crop Science, Ministry of Agriculture and Rural Affairs, Zhejiang University, Hangzhou 310058, China; zaidulhassan007@yahoo.com

**Keywords:** polyamines, maize, chilling stress, spermidine, gene expression

## Abstract

Chilling stress greatly inhibited the seed germination, plant growth, development and productivity in this study. The current research aimed to study the effects of different polyamine (PA) inhibitor combinations (Co), e.g., D-arginine (D-Arg), difluoromethylormithine (DFMO), aminoguanidine (Ag) and methylglyoxyl–bis-(guanyhydrazone) (MGBG) at different doses, i.e., 10 µM Co, 100 µM Co, 500 µM Co, 1000 µM Co and 1000 µM Co + 1 mM Spd (Spermidine) in two inbred lines of maize (*Zea mays* L.), i.e., Mo17 and Huang C, a sensitive and tolerant chilling stress, respectively. The combination treatments of PA inhibitors reduced the biosynthesis of putrescine (Put) in the tissues of both studied inbred lines. Application with 500 µM Co and 1000 µM Co did not result in a significant difference in Put concentrations, except in the coleoptile of Mo17. However, combining Spd to 1000 μM of PA inhibitors enhanced the Put, Spd, spermine (Spm) and total PAs in the roots, coleoptile and mesocotyls. Put and total PAs were increased by 39.7% and 30.54%, respectively, when Spd + 1000 µM Co were applied relative to their controls. Chilling stress and PA inhibitors treatments affected both inbred lines and resulted in differences in the PA contents. Results showed that enzymes involved in the biosynthesis of PAs (ornithine decarboxylase as ODC and S-adenosylmethionine decarboxylase as SAMDC) were significantly downregulated by 1000 µM Co in the tissues of both inbred lines. In contrast, the activity of PAO, a Pas degradation enzyme, was significantly improved by 1000 µM Co under chilling stress. However, Spd + 1000 µM Co significantly improved the activities of ODC and SAMDC and their transcript levels (*ODC* and *SAMDC2*). While it significantly downregulated the PAO activity and their relative genes (*PAO1*, *PAO2* and *PAO3*) under chilling stress. Overall, this study elucidates the specific roles of Spd on the pathway of PA inhibitors and PA biosynthesis metabolism in maize seed development in response to chilling stress. Moreover, the Huang C inbred line was more tolerant than Mo17, which was reflected by higher activities of PA biosynthesis-related enzymes and lower activities of PAs’ degradative-related enzymes in Huang C.

## 1. Introduction

Agricultural crops are confronted by a number of abiotic stress factors that limit their productivity [1,2,3,4,5]. Among abiotic stresses, chilling stress is one of the most severe stresses, which substantially inhibits the seed germination and seedlings development [6]. Low-temperature stress could reduce the growth and development of maize seedlings under field conditions. Maize has been reported to be sensitive to low temperature [7,8], especially at early growth stages, which results in the inhibition of seed germination and vegetative growth [9,10,11]. Previous studies reported that many physiological and metabolic processes of plants are repressed by chilling stress [12]. The tolerant level of maize plants depends on the hybrid type at both the chilling and recovery periods [7,13,14,15,16]. The exposure of chilling stress impairs numerous physiological and molecular functions of plants [12]. For instance, low-temperature stress can reduce the crop productivity through various actions such as reduction in plant growth rates [14], water uptake disturbance [15], photosynthesis efficiency [16], changes in membrane properties [17], extra production of reactive oxygen species (ROS) [18] and enzymatic and non-enzymatic antioxidants [19].

Increasing polyamine (PA) concentrations in plants during chilling stress could protect plant cells [20,21]. PAs have reported to be involved in several physiological and developmental processes in plants, such as survival of plant embryos and translation [22], cell proliferation, modulated gene expression and membrane stabilization [23,24]. Their substrates are present extracellularly and play a vital role in regulating the specific destined physiological processes such as lignification, oxidative burst, suberization and defense mechanisms [25]. The interaction of PAs (Spd and Spm) with plants and their mechanisms underpin the tolerance to environmental stresses and have been investigated in cereals and pulses at different aspects [26]. Polyamines (particularly Put, Spd and Spm) are involved in several physiological, metabolic and molecular mechanisms to regulate the plant growth and development under the abiotic stresses including chilling stress [27]. As such, PA catabolism pathway improved DAO and PAO activities, stimulating the generation of H_2_O_2_ molecules, which act as signaling molecules to improve antioxidant systems and alleviate oxidative damages [28]. Previous reports showed that PAs may alleviate chilling stress effects on the photosynthetic system due to their antioxidant properties [29] or their effect on the thylakoid membrane [30]. The key physiological and biochemical mechanism is that PAs may enhance plants adaptations to abiotic stress tolerance via their significant roles in osmotic regulation, inhibition in lipids peroxidation, stability and integrity of cellular membranes, scavenging oxidative stress, improvement in antioxidant enzymes and interaction with proteins and phytohormones biosynthesis [31]. Recently, several studies reported that seed priming with Spd substantially protected the cellular structures and alleviated the abiotic stress factors including salinity [32], heat [33] and chilling [34]. PAs can play important roles in improving the growth of several crop species under stress condition [35]. A previous study showed that Spd treatments have improved the seed vigor and growth of sweet corn as reflected by higher germination percentage, shoot height and dry weights of shoots and roots in comparison to their relative controls [36]. It had been reported that exogenous Spd at 0.01–1.0 mM concentrations improved alpha-amylase activity and seed germination in maize [37]. In addition, seed soaking with Spd was found to enhance the seed vigor and reduce the detrimental effects of chilling stress on the rice seeds, and thus enhance the chilling tolerance potential of rice seedlings [38,39].

PAs have modulated several biological functions such as cell division, differentiation and senescence, which enabled plants to cope with abiotic stress [40,41]. Additionally, they can stimulate cellular defense against oxidative damages through scavenging the free radicals induced by lipid peroxidation [42,43]. PAs play major roles in nitrogenous compounds’ metabolism in plants under normal as well as environmental conditions [44]. A recent study showed that the application of PAs to plants enhanced the shoot induction, root elongation and transformation efficiency in soybean [45]. A previous report stated that plants treated with exogenous Spd significantly increased endogenous Spd content, gibberellins and ethylene contents, while resulting in a reduction of ABA concentration in maize grain during seed development [37]. Moreover, changes in the transcription level of *ADC* genes and their activities were observed in *Brassica juncea* [46] and *Malus sylvestris* [47,48,49,50] under chilling stress. In chickpea genotypes, the activities of PA metabolism-associated diamine oxidase (DAO) and polyamine oxidase (PAO) enzymes as well as PA biosynthesis genes, such as arginine decarboxylase (*ADC*), spermidine synthase (*SPDS1* and *SPDS2*) and spermine synthase (*SPMS*), were upregulated under cold stress. Additionally, higher accumulation of Spd, Spm and Put contents, photosynthetic pigments and an elevated antioxidant defense system were more noticed in tolerant than sensitive genotypes under cold stress [28]. Similarly, the relative expression of the other PA-related genes such *SAMDC* were also affected in the saline condition in rice [51], soybean [52], wheat [53], Arabidopsis [54], apple [55] and maize [56]. PAs are oxidatively activated by the interaction of amine oxidases such as copper DAO and flavoprotein PAO. DAO reacts with Put and produces pyrroline, hydrogen peroxide and ammonia, while PAO reacts with Spd and Spm and produces pyrroline and 1,5-diabicyclononane, respectively, along with diaminopropane (Dap) and hydrogen peroxide. Both DAO and PAO enzymes are involved in PA biosynthesis, and their byproducts are involved in important physiological processes of plants under abiotic stress [57]. A previous study showed that cold stress decreased both transcripts in root tissues, while the accumulation of the *ZmMET1* transcript in the shoot mesocotyl was not affected by cold stress [58].

This study investigated the physiological changes in maize tissues by Spd under cold stress. Previous studies have only focus on the effect of PAs on alleviation of cold tolerance. In the present study, we focused on these effects beside their effects on the PA inhibitors and its signaling in the different tissues of maize. Hence, it is of great importance to analyze the content of PAs, enzymes involved in PA biosynthesis and their transcription levels under chilling stress for further understanding of the Spd-induced mechanism for enhancements to chilling tolerance and to provide insights for further analysis. Moreover, the genetic manipulation of the genes involved in PA biosynthetic pathways in different maize tissues may further elucidate the role of PA in plant developments under chilling stress. The present study hypothesized that the conversion of Spd to Put in the Spd-treated seedling in the presence of PA inhibitors and chilling stress. This statement supports the potential resources for the conversion of Spd to Put and can be used a physiological signal in maize under chilling stress. For this purpose, both sensitive and tolerant lines of maize to chilling stress were used as experimental materials under low temperature and sectioned into three tissues (root, mesocotyl and coleoptile). D-arginine (D-Arg), a competitive inhibitor of ADC and DFMO (difluoromethylornithine) and irreversible inhibitor of ODC, was used to inhibit the biosynthesis of Put. Aminoguanidine (Ag) was used to inhibit the degradation of Put. Methylglyoxyl–bis- (guanyhydrazone) (MGBG), a powerful inhibitor of SAMDC, was used to inhibit biosynthesis of Spd and Spm from Put [59]. The combinations of four PA inhibitors with different concentrations of exogenous Spd were used to investigate the potential roles of Spd in inhibiting the PA inhibitors’ effects in the root, mesocotyl and coleoptile and in the improvement in maize tolerance under chilling stress conditions.

## 2. Materials and Methods

### 2.1. Plant Materials and Growth Condition

Two inbred lines of maize cvs. Hung C (chilling tolerant) and Mo17 (chilling sensitivity) [60] were obtained from the Seed Center, College of Agriculture and Biotechnology, Hangzhou. All the seeds were stored at 4 °C before the seed germination test. The seeds were then sterilized for 5 min [61], then distilled water was used for seed washing (thrice) before the germination test. Thereafter, the sterilized seeds were germinated in darkness at 25 °C for 3 days in paper germination towels moistened with water. The normal seedlings with consistent growth were selected and incubated in Hoagland solution containing PA inhibitor combinations for 1 day at 25 °C, and then grown for 3 days at a low temperature (5 °C). Four PA inhibitors with different inhibitory functions were used together as a combination at different concentrations to check differential responses of treatments/inhibitors in both maize inbred lines. After that, the seedlings were transferred to constant temperature of 25 °C for 3 days as recovery period growth. For all of the growth period in the Hoagland solution, seedlings had a diurnal cycle of 12 h of light with luminous flux of 250 μmol m^−2^ s^−1^ and 12 h of darkness. Three replicates for each treatment and 50 seeds for each replicate were used in this study. Four polyamines’ inhibitors were used, e.g., D-arginine (D-Arg), difluoromethylormithine (DFMO) (an inhibitor for putrescine biosynthesis), aminoguanidine (Ag, an inhibitor for putrescine degradation) and methylglyoxyl–bis- (guanyhydrazone) (MGBG) (an inhibitor for spermidine, spermine and putrescine biosynthesis). The different concentrations of these PA inhibitors’ combinations were subjected to a preliminary screening test. The purpose is to use a combination of PA inhibitors to inhibit the production of Put, to the level that the content of Put does not decrease. After which the exogenous Spd was used to check the changes in Put content and whether Spd converts into Put. Therefore, in order to study whether Spd can convert into Put, a 1000 μM combination was used as a control, then 1mM Spd was applied on this basis (1 mM Spd + 1000 μM combination). The polyamine inhibitor combinations (Co.) in Hoagland solutions were used as follows:

(1) 10 µM Co. (10 µM D-Arg, 10 µM DFMO, 10 µM MGBG and 100 µM Ag);
(2) 100 µM Co. (100 µM D-Arg, 100 µM DFMO, 100 µM MGBG and 1000 µM Ag);
(3) 500 µM Co. (500 µM D-Arg, 200 µM DFMO, 500 µM MGBG and 5 mM Ag);
(4) 1000 µM Co. (1000 µM D-Arg, 200 µM DFMO, 1000 µM MGBG and 10 mM Ag);
(5) Spd + 1000 µM Co. (1 mM Spd + 1000 µM D-Arg, 200 µM DFMO, 1000 µM MGBG, 10 mM Ag) and the control of seedlings were without any treatment of polyamine inhibitors.

### 2.2. Polyamine Assay

PA concentrations in the roots, mesocotyls and coleoptile tissues of the maize seedlings were immediately measured after grown at low temperature (5 °C). Three replicates of 25 normal seedlings for each treatment were used for polyamines assay. Polyamine contents were estimated according to the method of [60]. Briefly, fresh samples (0.1 g) were homogenized with 1 mL of 5% (*w*/*v*) cold HClO_4_, kept for 1 h in ice and centrifuged for 30 min at 4 °C at 23,000× *g*. Then, the supernatant was stored at −70 °C for PA measurements. The PA standard samples for Put, Spd and Spm were prepared at different contents for the development of standard curves. Then the samples were subjected to HPLC, with 3.9 × 150 mm, 4 μm particle size reverse-phase (C18) column and a Waters 2487 dual λ absorbance detector.

### 2.3. Polyamine Biosynthesis Enzymes under Chilling Stress

The PA biosynthesis-related enzymes were estimated using the previous method [39]. For this purpose, 0.5 g for each the different tissues of maize (roots, mesocotyls and coleoptiles) from only the control, and 1000 µM Co. and Spd + 1000 µM Co.-treated plants were ground and homogenized with 3 mL of extraction buffer (pH 8.0) containing 25 mM potassium phosphate, 50 μM EDTA, 100 -M phenylmethylsulphonyl fluoride, 1 mM 2-mercaptoethanol and 25 mM ascorbic acid. The activities of ADC, ODC and SAMDC were determined according to the method of Lee et al. [62]. The reaction buffers for the ADC, ODC and SAMDC assays were 0.1 mL of 200 mM Tris-HCl (pH 8.5) buffer, 0.1 mL of 200 mM Tris-HCl (pH 8.0) buffer and 0.1 mL of 200 mM potassium-phosphate (pH 7.5) buffer, respectively. Then, the activities of ADC, ODC and SAMDC enzymes were determined by measuring the CO_2_ evolution. Spd synthase activity was estimated using the reported method of Kasukabe et al. [35].

### 2.4. Polyamines’ Degradative Enzymes under Chilling Stress

For polyamine oxidase (PAO) activity determination, 0.5 g of the tissues from only the control and 1000 µM Co. and Spd + 1000 µM Co.-treated plants were homogenized in 0.5 mL of 0.2 M phosphate buffer (pH 6.5) and centrifuged at 8000× *g* for 15 min at 4 °C [39]. The contents of H_2_O_2_ in grains were measured and expressed as mol g^–1^ DW [63].

### 2.5. Transcript Levels of PA Biosynthesis Genes

In order to analyze the transcription levels of PA biosynthesis genes, frozen tissues (200 mg) from only the control and from 1000 µM Co. and Spd + 1000 µM Co.-treated plants were ground thoroughly. Total RNA was extracted from the control and different treated seedlings by using RNA isolation. The concentration of the RNA was tested by NanoDrop [64]. cDNA was synthesized using Primer Script RT Reagent Kit from 1 µg of total RNA in a 20 µL reaction and diluted 4-fold with water. The primers used in real-time PCR (RT-PCR) are presented in Table 1. ACT1 was used as an endogenous control gene to normalize expression of the other genes. The PCR program was as follows: 30 s at 95 °C, followed by 40 cycles of 10 s at 95 °C and 30 s at 60 °C.

### 2.6. Statistical Analysis

Treatments were arranged in completely randomize design (CRD) using a factorial experiment. Before ANOVA, the data of the percentages were transformed according to y = arcsin [sqrt (x/100)]. All the results are the mean of three replicates ± standard deviation (SD) and all the data were subjected to an analysis of variance (ANOVA). When a significant (*p* < 0.05) F ratio occurred for treatment effects, a least significant difference (LSD) was calculated.

## 3. Results

### 3.1. Polyamine Concentration in Roots of Maize Seedlings

The mean data regarding the Put, Spd, Spm and total PAs in the root of both studied inbred lines as affected by the PA inhibitors and chilling stress are presented in Table 2. The contents of Put, Spd, Spm and total PAs were decreased in the seedlings exposed to chilling and PA inhibitors (Table 2). Under control conditions, Put, Spd, Spm and total PA contents were improved as compared to the PA inhibitor-treated seedlings. The lowest values of Put, Spd, Spm and total PAs were recorded at the exposure of 500 µM of PA inhibitor combinations. However, Spd combined with 1000 µM significantly improved the Put, Spd, Spm and total polyamines under chilling stress for both studied inbred lines. Moreover, the application with 10 µM was found to have the lowest negative effects on the Put, Spd, Spm and total PA contents in the maize roots as compared to 100, 500 and 1000 µM PA inhibitor treatments. The contents of Put and Spd in roots were higher in the Huang C inbred line under all PA inhibitor combinations and chilling stress, while the Spm contents were higher in Mo17 inbred line under the same treatments (Table 2). The concentrations of Spd in the seedlings treated with Spd + 1mM combinations were found to be lower than those observed in control seedlings. Moreover, Spd concentrations in the roots of Huang C were significantly decreased as the combination treatments were increased from 10 μM to 1000 μM. On the other hand, Spm concentrations in the roots of Mo17 were significantly decreased as the combination treatments were increased from 10 μM to 1000 μM, and the four combination treatments showed a significant decrease in Spm concentration relative to their controls (Table 2). The alterations’ trends in the values of total PA concentrations in the roots of Mo17 were similar to those of Put concentrations. It could be concluded that the Put and total polyamines were increased by 39.68% and 30.54%, respectively, by combining the Spd with 1000 µM relative to their controls.

### 3.2. Polyamine Concentration in Mesocotyl of Maize Seedlings

The application of PA inhibitor combinations during chilling stress notably decreased the Put, Spd, Spm and total PA contents in the mesocotyls of both inbred lines as relative to their controls (Table 3). The lowest values of Put (for only Mo17) and Spd (for both inbred lines) were noticed with 100 µM Co.-treated seedlings. While Spm contents were found lowest with 1000 µM Co.-treated seedlings. The combination of Spd with 1000 µM Co. PA inhibitors significantly improved the Put, Spd, Spm and total PAs, except for Spd and Spm contents in the Huang C inbred line, which showed lower values than those of 10 µM Co. of PA inhibitors (Table 3). The contents of Put and Spm in the mesocotyls of the Mo17 inbred line were higher under chilling stress and all PA inhibitor combinations, while the contents of Spd in Huang C were relatively higher under the same conditions (Table 3). Put concentrations in the mesocotyls of Mo17 that were treated by 100 μM, 500 μM and 1000 μM combinations displayed significantly lower range of values than those treated by 10 μM and Spd + 1 mM combinations and their controls (Table 3). The changes of Spm concentrations in the mesocotyl of Huang C were similar to those of Spd contents. Both 500 μM and 1000 μM combination treatments recorded significantly lower concentrations of total PA concentrations in the mesocotyls than those of the 10 μM and 100 μM combination-treated seedlings. There was a notable increase in Put (only in Mo17), Spd (only in Huang C), Spm (in both inbred lines) and total PAs (in both inbred lines) in the mesocotyl upon application with 10 μM as compared with other PA inhibitors (Table 3). It could be concluded that the applications of 10, 100, 500 and 1000 µM of PA inhibitors resulted in the following: the decrease of Put in the mesocotyls by 35.84%, 42.81%, 47.05% and 46.73%, respectively; the decrease of Spd by 46.91%, 60.32%, 63.11% and 65.01%, respectively; and the decrease of Spm by 33.86%, 60.81%, 78.54% and 90.60%, respectively, relative to their controls (Table 3).

### 3.3. Polyamine Concentrations in Coleoptile of Maize Seedlings

The PA concentrations in the coleoptiles as affected by PA inhibitors combination under the chilling stress are presented in Table 4. The results showed that Put, Spd, Spm and total PAs in both maize inbred lines were decreased in the seedlings treated with PA inhibitors as compared to their controls. Application with 1000 µM PA inhibitor combinations resulted in the lowest values of Put, Spd, Spm and total PAs as compared with other PA inhibitors. However, combining Spd with 1000 µM Co. significantly enhanced the Put, Spd, Spm and total PA contents (Table 4). The contents of Put and Spm in coleoptiles were notably higher in Mo17 under chilling stress and PA inhibitor combinations, while Spd contents were higher in Huang C under the same conditions, except in the 500 µM Co.-treated Mo17 inbred line (Table 4). Application with the 10 µM PA inhibitor resulted in an improvement in Put, Spd, Spm and total PA contents in coleoptiles of both inbred lines, except for Put in Huang C, which was found to be higher in the plants treated with 100 µM of PA inhibitors as compared with other PA inhibitors. It can be concluded that 10, 100, 500 and 1000 µM of PA inhibitors resulted in the following: the reduction of Put in the coleoptiles by 68.41%, 68.72%, 73.09% and 73.92%, respectively; the reduction of Spd by 21.76%, 63.20%, 48.22% and 57.61%, respectively; and the reduction of Spm by 57.88%, 85.18%, 62.95% and 85.65%, respectively, relative to their controls (Table 4).

### 3.4. Polyamine Biosynthetic and Degradative Enzymes under Chilling Stress

**Figure 1:**
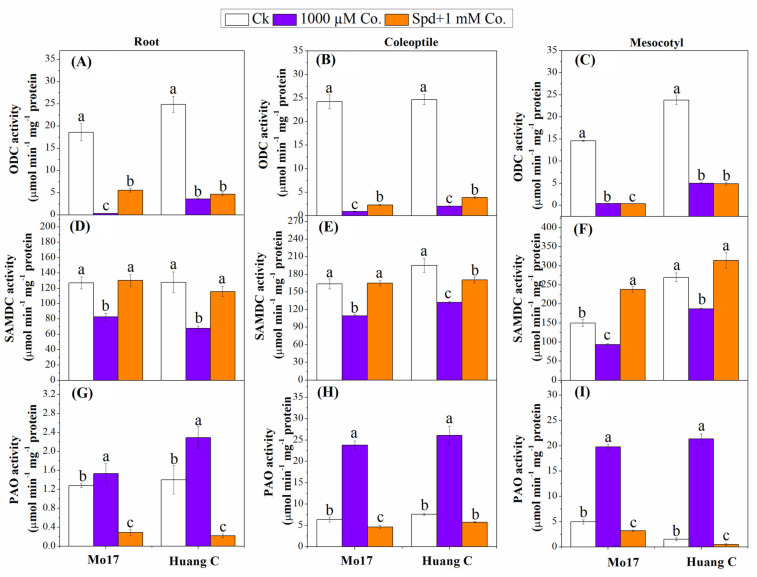
Effects of polyamine inhibitor combinations on ODC activity (**A**–**C**), SAMDC activity (**D**–**F**) and PAO activity (**G**–**I**) in the root, coleoptiles and mesocotyls of two maize inbred lines under chilling stress. Different letters mean significant difference at *p* ≤ 0.05.

The enzymes involved in PA synthesis (ODC and SAMDC) and polyamines’ degradation (PAO) as affected by the PA inhibitors and chilling stress are illustrated in Figure 1. The highest activity of ODC was observed under control condition as compared with 1000 µM Co. (Figure 1). The activity of ODC was significantly decreased by 1000 µM Co. in both maize inbred lines as compared with the control condition (Figure 1A–C). The combination of Spd with 1000 µM Co. noticeably affected the ODC activity in roots and coleoptiles (Figure 1A,B), while there were no significant effects observed in mesocotyls in the Huang C inbred line (Figure 1C). The activity of SAMDC was significantly decreased in roots, coleoptiles and mesocotyls at the exposure of 1000 µM Co. as compared to controls and Spd + 1000 µM Co. treatments (Figure 1D–F). There was no significant difference between controls and Spd + 1000 µM Co. treatments except in the coleoptiles of Hang C inbred line that showed significant differences. The incorporation of Spd with 1000 µM Co. PA inhibitors significantly improved the activity of SAMDC in roots, coleoptiles and mesocotyls in both maize inbred lines (Figure 1D–F). The activity of PAO was greatly increased in roots, coleoptiles and mesocotyls of both maize inbred lines when 1000 µM Co. was applied under chilling stress (Figure 1G–I). However, there was a significant decrease in PAO activity on plants that were treated with the combined of Spd and 1000 µM PA inhibitor combinations.

### 3.5. Polyamines Biosynthesis Genes in Response to PA Inhibitors under Chilling Stress

**Figure 2:**
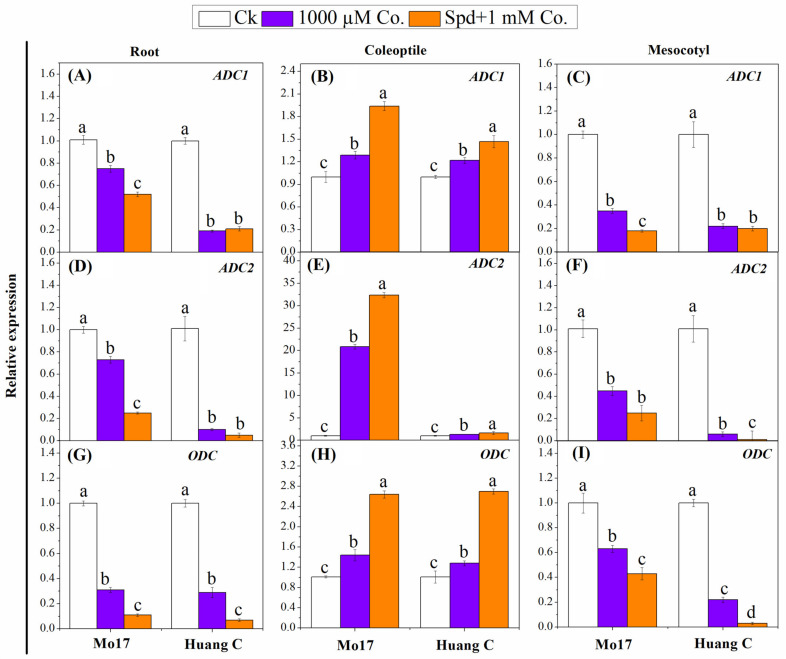
Effects of polyamine inhibitors combination on the relative expression of *ADC1* (**A**–**C**), *ADC2* (**D**–**F**) and *ODC* (**G**–**I**) in the root, coleoptiles and mesocotyls of two maize inbred lines under chilling stress. Different letters mean significant difference at *p* ≤ 0.05.

In order to reveal the molecular mechanisms by which PAs could help maize plans to cope with chilling stress, the PA biosynthesis genes were determined. The results from Figure 2 indicated the relative expression of *ADC1*, *ADC2* and *ODC* genes in different tissues of both maize inbred lines under PA inhibitors and chilling stress. The expression level of *ADC1* gene was significantly downregulated in roots and mesocotyls of both inbred lines when 1000 µM Co. and Spd + 1000 µM Co. were applied (Figure 2A–C). Interestingly, the expression of *ADC1* gene was upregulated in the coleoptile of both inbred lined under the same conditions (Figure 2B). The expression level of *ADC1* was found to be higher in the roots and mesocotyls of Mo17 inbred line when treated with 1000 µM Co PA inhibitors as compared with Spd + 1000 µM Co. treatment. A similar trend was observed in case of *ADC2* expression in both inbred lines under the same conditions (Figure 2D–F). The expression level of *ADC2* was significantly upregulated in the roots of Mo17 inbred line and in the mesocotyls of Huang C inbred upon being treated with 1000 µM Co PA inhibitors as compared to being treated with Spd + 1000 µM Co. treatment. The relative expression of *ODC* in the roots and mesocotyls was found to be higher in control seedlings and significantly downregulated under 1000 µM Co. and Spd+1000 µM Co treatments (Figure 2G–I). On the other hand, *ODC* displayed a significant upregulation in the coleoptiles of both inbred lines against 1000 µM Co. and Spd + 1000 µM Co. treatments (Figure 2H).

**Figure 3:**
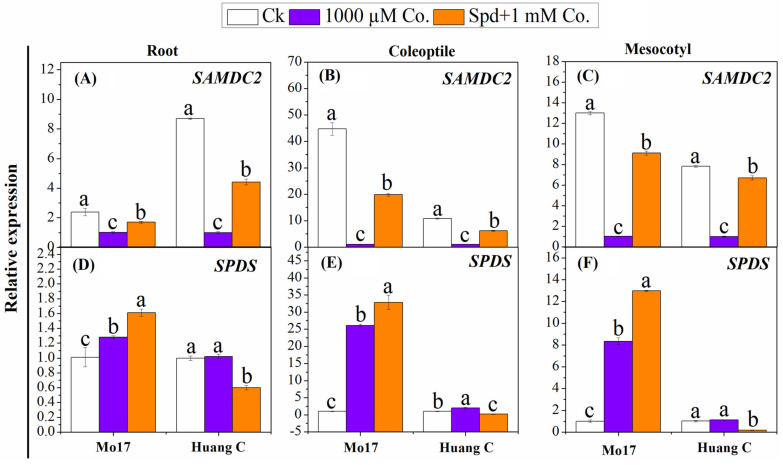
Effects of polyamine inhibitors combination on the relative expression of *SAMDC2* (**A**–**C**) and *SPDS* (**D**–**F**) in the root, coleoptiles and mesocotyls of two maize inbred lines under chilling stress. Different letters mean significant difference at *p* ≤ 0.05.

The relative expression level of *SAMDC2* genes in studied plant tissues of both inbred lines were significantly downregulated when 1000 µM Co. was applied under chilling stress (Figure 3A–C). The expression levels of *SAMDC2* were higher in both maize inbred lines treated with Spd + 1000 µM Co. as compared with plants treated with the 1000 µM PA inhibitor alone. Higher expression levels of *SAMDC2* genes in maize tissues were observed under the control conditions. In contrast, the relative expression levels of *SPDS* were significantly upregulated in the seeds treated with both 1000 µM Co. and Spd + 1000 µM Co., especially in the Mo17 inbred line (Figure 3D–F). The highest expression level of *SPDS* in all maize tissues was observed with Spd + 1000 µM Co. treatment of the Mo17 inbred line. Interestingly, similar treatment resulted in the lowest expression level of *SPDS* in the tissues of the Huang C inbred line (Figure 3D–F). Application of the 1000 µM PA inhibitor alone recorded a higher expression level of *SPDS* in roots, coleoptiles and mesocotyls of the Huang C inbred line as compared with Spd + 1000 µM Co. treatment.

**Figure 4:**
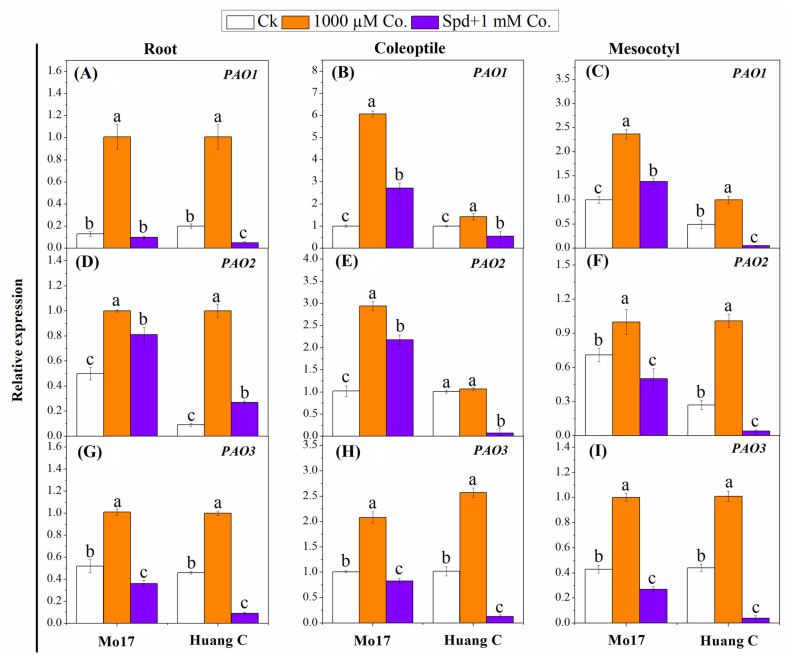
Effects of polyamine inhibitors combinations on the relative expression of *PAO1* (**A**–**C**), *PAO2* (**D**–**F**) and *PAO3* (**G**–**I**) in the root, coleoptiles and mesocotyls of two maize inbred lines under chilling stress. Different letters mean significant difference at *p* ≤ 0.05.

Mean data regarding the relative expression of the enzyme involved in the PAs’ degradative enzymes are presented in Figure 4. The relative expressions of *PAO1*, *PAO2* and *PAO3* genes in the targeted tissues of both maize inbred lines were significantly upregulated in the seedlings that were treated with 1000 µM Co. as compared to the relative controls (Figure 4). Combining Spd with 1000 µM Co. resulted in the downregulation of these genes under chilling stress. The lowest expression levels of *PAO1*, *PAO2* and *PAO3* were observed with Spd + 1000 µM Co. in the root of Mo17, while the lowest expression levels of these genes were noticed in the coleoptiles and mesocotyls of Mo17 inbred line (Figure 4).

## 4. Discussion

Abiotic stresses specifically chilling stress have severely affected the plant growth and development globally [65,66]. Generally, plants with high-stress tolerance have differentially enhance PA biosynthesis in response to chilling stress [67]. PAs play crucial roles for in abiotic stress adaptation, which might be due to their roles in osmotic regulation, inhibition in lipid peroxidation and scavenging oxidative stress by improving antioxidant enzymes and inducing hormones and ethylene signaling [68]. Moreover, the inhibitor combination treatments could also regulate the interaction between the different PA compositions under chilling stress, leading to enhancement in seedlings’ growth and development [69]. In the present study, the chilling sensitive Mo17 did not recover after the recovery period under normal temperature in the seedlings treated with the 1000 μM combination inhibitor. However, the cold-tolerant Huang C showed a reverse trend under the same conditions and inhibited the growth reduction. However, the growth of Mo17 was found to be recovered upon being treated with the Spd + 1mM combination, while it was not effective for the recovery of Huang C. It could be stated that Spd had better growth recovering effects on maize chilling-sensitive inbred lines than cold-tolerant inbred lines under low chilling stress.

PAs are endogenous plant growth regulators that can stimulate plant growth and development under different abiotic stresses [70,71,72]. Several studies have reported that PAs contributed to the regulation of plants’ responses to various environmental constrains through binding the membrane phospholipids, scavenging free radicals by increasing the antioxidant enzymes’ activities and osmotic adjustment [73,74]. In the present study, two maize inbred lines were treated with four types of PA inhibitors under chilling stress. The concentrations of Put, Spd, Spm and the total PAs in different tissues of maize seedling were obviously declined under PA inhibitor treatments. These findings stated that the PA inhibitors can be accumulated causing inhibition in the synthesis of PAs. Moreover, there was no significant difference in Put concentration between 500 μM and 1000 μM combination treatments in all tissues of both inbred lines, except in the coleoptiles of Mo17. Both Mo17 and Huang C inbred lines showed different patterns in the accumulation of PAs under both chilling stress and the PA inhibitor treatments. As such, the authors of [75] reported that differences in genotypes to stress have displayed diverse degrees of PAs’ accumulation under abiotic stresses. Strikingly, previous reports stated that higher contents of Spd and Spm were synthesized by tolerant species, while a higher concentration of Put was synthesized by the sensitive species of the same plant variety under the same stress conditions [76,77,78]. In the present study, Put concentrations in the tissues of both inbred lines’ seedlings treated with the Spd + 1mM combination were significantly higher than those in the seedlings treated with the 1000 μM combination treatments, suggesting that Spd can be converted to Put and thus enhance chilling stress tolerance. Moreover, the results the conversion efficiency of Spd to Put in the root were found to be higher than those found in mesocotyls and coleoptiles. Probably, PAs carried out more transport distance to the roots than shoots. The present study is consistent with the previous study by Moschou et al. [79] in tobacco (*Nicotiana tabacum*), indicating that the Spd and Spm synthesis was higher in the shoot, while Put synthesis was more in roots. This demonstrates the significant increase in root viability, length and root mortality during germination in the seeds primed with exogenous Spd under water stress [80]. A similar study also by Xu et al. [81] reported that Put treatment was more effective for tobacco roots than shoots during seed germination under chilling stress. A recent study showed that Spd stimulated the phytohormones’ metabolisms, such as GA and ethylene, and simultaneously inhibited ABA biosynthesis, leading to improved seed germination and vigor of maize seeds [36]. In the present study, Put, Spd, Spm and total polyamine contents in the different maize tissues were significantly improved by the application with Spd and 1000 µM Co. of PA inhibitors as compared with the PA inhibitors alone. This might be due to the capability of Spd treatment to improve Spd uptake and SAMDC activity and to simultaneously reduce PAO activity, and consequently, reduce the PA biosynthesis-related enzymes [82]. Interestingly, the application with Spd along with PA inhibitor combinations showed different patterns for the PA enzymes’ activities and their transcript levels in the different maize tissues; this might be reflecting a different feedback regulation of Spd under chilling stress. The present study showed that the PA inhibitors cause a significant reduction in the PA contents, PA biosynthesis-related enzymes and their transcript levels; this might be due to increasing of the cell membrane integrity with the application of PA inhibitors [36]. However, the application of the PAs diminished the detrimental effects of PA inhibitors through alleviation the oxidative damage in the plants by scavenging the free radicals [39].

Put and Spd concentrations were highly increased upon being treated with the Spd and 1 mM combination as compared with those treated with the 1000 μM combination. This statement supports the potential resources for the conversion of Spd to Put and can be used a physiological signal in maize under chilling stress. These results are consistent with the previous study, reporting that the conversion efficiency of Spd to Put might be related with the absorption and transportation of Spd in seedlings tissues [83]. The present study stated that the differences in the absorption and transform of Spd may vary according to the genotype tolerance to chilling stress, and it seemed that a higher Spd concentration was absorbed and, simultaneously, a higher Put concentration was produced from Spd to improve the cold resistance in chilling-sensitive Mo17. These findings are consistent with the results under normal temperature conditions [84]. However, De-Agazio et al. [85] suggested that Spd from did not convert to Put regardless of the plant growth condition. However, the chemical and physiological processes underlying the Spd conversation to Put in more plants need to be further studied.

In the present study, Spd treatments inhibited the PA inhibitors’ effects and simultaneously improved the PA biosynthesis-related enzymes such as ADC, ODC, SAMDC and Spd synthase under chilling stress conditions. These enzymes have differential responses to the different stresses; as such, ADC is more specifically responsive to environmental stress, while ODC is more responsive to any kind of stress [30,39]. Similarly, ADC contributed to increased Put concentrations under abiotic stress [86]. In the present study, an increase in PAO activity, an enzyme involved in the PAs’ degradation, was observed in the plants treated with the PA inhibitors under chilling stress, which was diminished by Spd applications. These results are in line with the findings of Amini et al. [28], who observed an increase in PAO activity under cold stress, which might be due to the reduction in Put and Spd levels. Our previous study also reported that the increase in SAMDC activity resulted in escalated uptake of Spd, and thus enhanced the chilling tolerance of rice cultivars [39].

In order to investigate the molecular mechanisms of PA biosynthesis in response to chilling stress further, the transcript levels of their genes were analyzed in the present study. The genetic manipulation of crop plants with genes encoding enzymes of PA biosynthetic pathways may provide better stress tolerance to crop plants [86]. These genes have been investigated under different environmental stresses and at different growth stages of several plant species [49,52,53]. In the present study, the Spd-treated seedling resulted in the upregulation of *ADC1*, *ADC2*, *ODC* and *SAMDC2* transcripts and *SPDS*, and decreased the relative expression of *PAO1*, *PAO2* and *PAO3* genes, irrespective of the chilling stress and PA inhibitors. These genes were also upregulated in rice seedlings exposed to chilling stress [39]. Previously, the expression levels of *ADC2* gene were downregulated by the induction of *ODC1* gene under salinity stress, suggesting that the involvement of *ADC* pathway for the PA biosynthesis in higher plants under abiotic stresses [87]. Similarly, a significant increase in transcript levels of *ADC*, *SPDS1*, *SPDS2* and *SPMS* genes was observed in the chilling-tolerant and chilling-sensitive genotypes, respectively, whereas the expression of *ODC* genes decreased significantly in all genotypes under chilling stress [28]. Another study has reported that the relative expressions of *ADC2* genes were upregulated in *Arabidopsis thaliana* and rice [47,88]. Moreover, overexpression of the Spd synthase gene in transgenic *Arabidopsis thaliana* maintained higher levels of Spd contents and enhanced the plant tolerance to chilling, suggesting that Spd plays an important role as an osmolyte regulator in stress signaling pathways, which boosts stress tolerance mechanisms in plants under stress conditions [35]. The decrease of PAs might be due to the increasing conversion rate of SAM to dcSAM by increasing the transcript levels of *SAMDC2*, leading to efficient conversion that resulted in the reduction of Put and Spd levels [89]. These *PAO* genes have been investigated in different plant species such as *A. Thaliana* [90], tobacco [91], rice [92], barley [93] and maize [94].

## 5. Conclusions

In conclusion, chilling stress can reduce the polyamine (PA) contents through decreasing the activities of enzymes taking part in PA biosynthesis and increasing the degradation of the PAs through the PAO enzyme in both maize inbred lines. The application of all studied PA inhibitor combinations resulted in the reduction of PA biosynthesis and increase of polyamines’ degradation. The PA inhibitors in combination with Spd treatments enhanced the levels of PA contents, enzymes related to PA biosynthesis and their relative gene expressions in roots, coleoptiles and mesocotyls under chilling stress. Both inbred lines showed different patterns to the biosynthesis and degradation of PAs under chilling stress and a supply of PA inhibitors. The enhanced seed development of both maize inbred lines under the PA inhibitors and chilling stress is closely associated with the elevated biosynthesis of Spd, Spm and Put in maize tissues. This suggested that Spd can maintain the balance between the activation and degradation processes of PA biosynthesis by controlling related enzymes’ activities as well as transcription levels under chilling stress. The present study confirmed the involvement of PAs in the regulation of growth, development and chilling tolerance of maize seedlings, which may have implications for maize cultivation in low-temperature conditions, although field investigations are prerequisites to broaden laboratory findings.

## Figures and Tables

**Table 1:** Real-time PCR primers for genes expressions detection.

Locus	Primer Name	Primer Orientation	Sequence (5′–3′)
*NC_008332.1*	18s rRNA	Forward	ACATGCGCCTAAGGAGAAATAG
	18s rRNA	Reverse	ACCTCCATGCTCACTGGTACTT
*NM_001323076*	ZmADC1	Forward	GCTACGGCTCAAGGTACCAG
	ZmADC1	Reverse	CCGAACTCCACAATGTCCTC
*NM_001138726*	ZmADC2	Forward	GGAGCCACTCATGACCAAAG
	ZmADC2	Reverse	CAGGGACCTTGTATTCGTTGA
*NM_001148682*	ZmODC	Forward	GCGCCTACTCCACAGGTTC
	ZmODC	Reverse	CGTAGATCTTAATCTCCGACGTG
*NM_001155838*	ZmSPDS	Forward	TGTTCAATTCCATCCCCTAAA
	ZmSPDS-R	Reverse	TCCACTGAACTGTGTCTGGAA
*NM_001112243*	ZmSAMDC2	Forward	TGTGGCTACTCCATGAATGC
	ZmSAMDC2	Reverse	CGTAACTGGCGTAGCTGAAA
*NM_001111636*	ZmPAO1	Forward	CCAGCAGCAGGAGAGGTTAC
	ZmPAO1	Reverse	GCGTCAGGGTACTGCTTCTC
*NM_001329439*	ZmPAO2	Forward	CACACACACCATCCGCTATT
	ZmPAO2	Reverse	CATCAGCAGCAGCAAGACC
*XM_008652490*	ZmPAO3	Forward	AAAGCCACACACACCATCTG
	ZmPAO3	Reverse	CAGCAGCAGCAAGACCTGTA

**Table 2:** Effect of polyamine inhibitor combination treatments on polyamine concentrations of the root (n mol/g FW) under low-temperature stress.

Treatment	Put		Spd		Spm		Total Polyamine	
	Mo17	Huang C	Mo17	Huang C	Mo17	Huang C	Mo17	Huang C
0 (CK)	639.5 b	673.3 b	178.7 a	302.9 a	23.6 a	13.6 a	841.8 b	989.8 b
10 µM Co.	551.7 c	559.9 c	100.9 c	289.1 c	17.2 b	9.8 b	669.8 c	858.7 c
100 µM Co.	311.6 d	424.8 d	85.4 c	282.0 c	11.8 c	1.4 d	408.8 d	708.1 d
500 µM Co.	239.6 e	358.3 e	92.5 c	194.7 d	4.4 d	0.0 e	336.4 e	553.0 e
1000 µM Co.	243.0 e	356.4 e	94.0 c	155.4 e	0.0 e	0.0 e	337.0 e	511.8 f
Spd + 1 mM Co.	1269.7 a	905.6 a	129.8 b	312.1 a	13.5 c	6.4 c	1412.9 a	1224.1 a

Different letters following the data within each column mean significant difference at *p* ≤ 0.05.

**Table 3:** Effect of polyamine inhibitor combination treatments on polyamine concentrations of mesocotyl (n mol/g FW) under low-temperature stress.

Treatment	Put		Spd		Spm		Total Polyamines	
	Mo17	Huang C	Mo17	Huang C	Mo17	Huang C	Mo17	Huang C
0 (CK)	1369.8 a	738.4 a	195.8 a	245.0 a	25.0 b	31.4 a	1589.6 a	1018.6 a
10 µM Co.	953.7 c	398.9 c	107.0 d	127.9 b	24.5 b	12.8 b	1085.2 c	539.7 c
100 µM Co.	805.0 d	400.4 c	99.5 e	75.4 d	13.3 c	8.8 d	917.8 d	484.6 d
500 µM Co.	781.2 d	335.0 d	111.2 c d	51.4 e	8.5 d	3.6 e	900.9 d	390.0 e
1000 µM Co.	790.9 d	332.4 d	115.1 c	39.1 f	3.2 e	2.10 f	906.0 d	371.5 e
Spd + 1 mM Co.	1166.0 b	444.3 b	161.5 b	117.6 c	41.4 a	11.5 c	1368.9 b	573.3 b

Different letters following the data within each column mean significant difference at *p* ≤ 0.05.

**Table 4:** Effect of polyamine inhibitor combination treatments on polyamine concentrations of coleoptile (n mol/g FW) under low-temperature stress.

Treatment	Put		Spd		Spm		Total Polyamines	
	Mo17	Huang C	Mo17	Huang C	Mo17	Huang C	Mo17	Huang C
0 (CK)	2044.7 a	932.9 a	535.3 a	619.9 a	80.5 a	81.2 a	2660.5 a	1634.0 a
10 µM Co.	620.5 c	319.7 c	439.4 b	464.4 b	51.4 b	16.7 c	1111.3 b	800.6 b
100 µM Co.	604.3 c	326.4 c	338.4 c d	390.8 c	43.4 c	12.9 d	986.1 c	730.1 c
500 µM Co.	556.2 d	244.6 d	363.5 c	235.2 f	46.3 c	13.6 d	965.9 c	493.3 d
1000 µM Co.	529.0 e	247.0 d	231.6 e	258.0 e	13.9 e	9.3 e	744.4 d	514.3 d
Spd + 1 mM Co.	778.4 b	369.0 b	315.0 d	314.2 d	31.7 d	32.7 b	1125.0 b	715.8 c

Different letters following the data within each column mean significant difference at *p* ≤ 0.05.

## Data Availability

Not applicable.
